# An Interesting Letter from New York

**Published:** 1884-11

**Authors:** Hunter P. Cooper

**Affiliations:** Presbyterian Hospital, New York


### AN INTERESTING LETTER FROM NEW YORK.

---

### DIAGNOSIS OF CANCER OF THE STOMACH—SOUTHERN PHYSICIANS IN NEW YORK—ACADEMY OF MEDICINE, ETC.

---

*Editors Atlanta Medical and Surgical Journal:*

I have not been unmindful of my promise to write an occasional letter for your interesting journal, but from a medical standpoint, New York is absolutely devoid of interest during the summer. Hence I postponed my first letter until the great medical luminaries should return from their vacation. Each fall some of our noted professors come from the medical centres of Europe with a number of new ideas. These ideas are intended to be used as certain aids in establishing the diagnosis of obscure diseases, or else they are to revolutionize the treatment of, and render amenable, diseases which have hitherto baffled the physician's skill. It is needless to say that in the majority of cases these wonders fail to work on this side of the Atlantic. One of the latest of these "wrinkles" was born in Paris and claims Dr. Dujardin-Beaumetz for its distinguished father. It is, that in cancer of the stomach and liver, the amount of urea excreted daily never exceeds one hundred and fifty grains.

As I have had under observation for several months a man advanced in life, who suffers from intense and almost constant pain in the region of the stomach, nausea and vomiting, marked cachexia and loss of strength, I concluded to apply this crucial test. Analysis showed that on four different days he excreted 180, 345, 352, and 240 grains of urea. This excessive excretion of urea might have shaken my faith in the diagnosis of cancer of the stomach, but for the fact that physical examination reveals a large tumor half as large as a man's fist, two inches to the right of the median line, and just below the free border of the ribs.

Of course the diagnosis may be incorrect, but in view of all the symptoms, nothing short of an autopsy will disprove it.

In two other cases, giving the same symptoms plus vomiting of blood, I found in the urine of 24 hours 244 and 369 grains of urea respectively. In one of these cases, however, no tumor has as yet been discovered. In the quantitative test for urea, I invariably use Squibb's method, on account both of the simplicity of application and the freedom from sources of error, as compared with other methods.

Another new aid to diagnosis of cancer of the stomach is founded on the fact(?) that in this disease hydrochloric acid is at all times absent from the viscus. The test is applied as follows: The patient is made to swallow a gelatine capsule, containing a piece of clean sponge with a strong silk thread tied around it, the thread being brought through the end of the capsule. The sponge is allowed to remain in the stomach half an hour, and then the doctor hauls it up by means of the silk thread hanging from the patient's mouth. The sponge is then tested for hydrochloric acid by means of a solution of tropæolin.

This method I have seen applied only once, and hence have not formed any positive opinion as to its value.

There is still another recent discovery which promises vastly more practical results. It consists in the employment of cocaine hydrochlorate to produce local anæsthesia in operations on the eye. This substance is a salt derived from erythroxylon coca. Several drops of a 2 per cent. solution are dropped into the eye and this is repeated in a minute or two. In five minutes complete anæsthesia and analgesia of the eye are produced, and the effect lasts about half an hour.

Dr. Agnew, of this city, is now trying this method, and so far, with excellent results. There is a brilliant future for cocaine should subsequent investigations confirm its value.

Dr. Lawson Tait, the celebrated English ovariotomist, performed two ovariotomies at Bellevue Hospital last month in the presence of eight hundred practitioners and students. He uses none of the elaborate antiseptic paraphernalia which so complicate the operation in New York hospitals. The rapidity with which he operates is something wonderful. In each case he had concluded the operation and stitched up the abdominal wound in less than twenty minutes. A few days ago the house physician of Bellevue informed me that in neither case had there been an untoward symptom since the operation (three weeks).

Post-graduate study in New York has received a new impetus from the establishment of two schools for practi ioners. This feature will soon render it superfluous for a man to seek clinical instruction abroad. In one of these schools, the polyclinic, two of the professors are representative Southern men. One of these is Dr. Gibney, Professor of Orthopedic Surgery, who was born and raised in old Kentucky. He is still a young man, but is already a recognized authority in his specialty. The other is Dr. Wyeth, of Alabama, who has a growing reputation as an anatomist and surgeon. One is surprised at the large number of Southern physicians located in New York, and what is more gratifying, very many of them are men of note.

I will close my letter with a brief outline of the discussion at the last meeting of the New York Academy of Medicine, October 16th. The paper of the evening, by Dr. Ambrose L. Ranney, Professor in the University Medical College, had for its title "The Therapeutical Effects of the Internal Administration of Hot Water in Nervous Diseases."

Dr. Ranney said the dose to begin with should be from one to one and a half goblets at a temperature ranging between 110° and 150° F. The dose should be taken with great regularity an hour and a half before each meal, and just before going to bed at night. The temperature of the water can gradually be increased as the patient becomes accustomed to it. This plan must frequently be kept up for six months before its good effects are apparent. The size of the dose must be regulated by the daily amount and specific gravity of the urine. During the hot water treatment cold beverages must be interdicted.

Effects of the Treatment—1st. It increases downward peristalsis. 2d. It promotes cutaneous circulation and increases perspiration. 3d. It increases the quantity and solid ingredients of the urine. 4th. It stimulates the liver and pancreas to secretion.

### THEORY OF THE ACTION OF HOT WATER.

He adopts the neurotic theory, and believes that the nerves of the stomach and solar plexus are directly influenced, and all the other viscera indirectly, through the agency of the sympathetic system.

He then cites cases in which he has used it with great advantage. One patient who was subject to severe and frequent attacks of facial neuralgia was nearly cured. In a case of locomotor ataxia, with marked diplopia, the patient required the assistance of a body servant and two canes in order to walk. Under the hot water treatment diplopia disappeared, and locomotion greatly improved. Three cases of severe and long-continued gastralgia were cured. The urine of a diabetic patient still contained a large quantity of sugar, in spite of a strictly non-sacharine diet. He was put on the hot water treatment, and the sugar rapidly disappeared. Several cases of nerasthenia with gastric derangement had been cured.

In conclusion, he urges a trial of this method, for the following reosons:

1st. It is perfectly harmless.

2d. Its effects are uniform.

2d. It does not interfere with other modes of treatment.

4th. It promotes cutaneous circulation.

5th. It benefits certain chronic nervous diseases accompanied with defective assimilation of food.

6th. It influences the vaso motor system in functional nervous diseases.

Dr. E. C. Sequin was then called on by the President to discuss the paper.

Dr. Seguin said he had had very little experience with the hot-water treatment. He did not feel sure but that the good effects were due, first, simply to the *water* itself, independent of its temperature; or, second, to the diet prescribed by Dr. Ranney in conjunction with the hot water. He had used water in the same class of cases mentioned by Dr. Ranney, but he ordered it to be drank with the meals, and at any temperature the patient preferred.

Dr. Amidon stated that he had used the hot water with much benefit in gout and rheumatism. He found that it increased the quantity of the urine and the amount of urea, chloride of sodium, and phosphoric acid contained therein, hence it favored tissue metamorphosis.

Dr. Birdsall said he regarded Dr. Ranney's cases as proving nothing at all, in as much as Dr. R. was administering other remedies at the same time. He believed that the good effects in functional nervous diseases are largely due to the *heat.*

Dr. Dana stated that he had used the hot-water treatment only in hysterical and extremely nervous women, who suffered from gastric derangement, but he had been disappointed in the results, for although it relieved the symptoms considerably, it did not cure. He had never, however, used it as systematically as Dr. Ranney advised.

Dr. Ranney again took the floor and answered the criticisms in an able manner.

The meeting then adjourned. HUNTER P. COOPER.

*Presbyterian Hospital, New York.*

